# Methane and Constipation-predominant Irritable Bowel Syndrome: Entwining Pillars of Emerging Neurogastroenterology

**DOI:** 10.7759/cureus.4764

**Published:** 2019-05-28

**Authors:** Syed Hamza Bin Waqar, Aiman Rehan

**Affiliations:** 1 Internal Medicine, Civil Hospital Karachi, Karachi, PAK; 2 Internal Medicine, Dow University of Health Sciences, Karachi, PAK

**Keywords:** c-ibs, irritable bowel syndrome (ibs), lovastatin, methaninobrevibacter, dysbiosis, neurotransmission, methane, intestinal transit, constipation

## Abstract

Functional gut disorders have long known to cause depravity in quality of life. Among the group of these heterogeneous disorders, irritable bowel syndrome (IBS) has been known to affect a large chunk of our population. IBS is not as simple as it sounds. Caused by a multitude of factors, the heterogeneity of this disorder has laid the foundation for research and the new principles of neurogastroenterology. Dysbiosis and methane production are one of the forthcoming factors that are currently under investigation. Down the road of exclusive enteric anaerobic fermentation of polysaccharides, methane is produced. It was considered to be an inert gas in the past, with little to no role in gut activity but now it is established that it has an impressive role in the etiology of constipation-predominant IBS (C-IBS). Acting as a neurotransmitter, it is known to affect ileal and colonic transit time, which has currently been shown in animal studies. Many laxatives, ionophore antibiotics, drugs like rifamixin and neomycin have been targeted against this very principle. Lately, lovastatin has emerged as a potential pharmacologic therapy to devoid the gut of methane without disrupting the gut niche in itself and has shown promise in relieving the symptoms of C-IBS. The goal of this article is to compile and assemble the literature available on IBS and the neuromodulation of methane to teach physicians and research scientists about the current age of gastroenterology and the growing need to emphasize the role of methane in the symptomatology of functional gut disorders like C-IBS.

## Introduction and background

Irritable bowel syndrome (IBS) is an idiopathic clinical entity characterized by chronic recurrent abdominal pain in relation to altered bowel habits, comprising constipation or diarrhea. The current consensus of Rome IV 2016 defines IBS as a functional syndrome having abdominal pain with two of the three features: (a) related to defecation, (b) associated with the change in frequency of stool, or (c) associated with the change in the form or appearance of stool. It is also important that symptoms of abdominal pain should be present on an average of at least one day per week [1]. As simple as it may sound, IBS doesn't always follow a well-defined route and may present in a variety of ways, at times mimicking organic disorders like lactose intolerance, which then need to be properly excluded [2]. IBS is of three main subtypes: diarrhea-predominant IBS (D-IBS), constipation-predominant IBS (C-IBS), and mixed IBS (M-IBS). Patients fluctuate in between the various phenotypes of this disorder mainly between C-IBS or D-IBS with M-IBS [3].

Dysbiosis, an altered gut-brain axis, increased intestinal permeability, and visceral hypersensitivity has all been documented as potential host factors along with environmental changes contributing to the etiopathogenesis of IBS [4]. C-IBS, in particular, is associated with changes in gut microbiota, especially at the molecular level [5].

Shocking as it may seem, patients with C-IBS have a distinct gut ecology. In particular, they have more methanogens that belong to the Archaea group of bacteria in contrast to D-IBS, which strikingly lack these organisms. These microbiotas generate methane gas from the fermentation of endogenous and exogenous carbohydrates. Methane, which was long considered to be an inert gas, is now known to behave as a neurotransmitter that has paved the path for pharmacologic therapy to create novel drugs targetting methanogenesis, in particular, to control C-IBS [6-7]. This unique correlation of irritable bowel syndrome with methane makes it a center of attraction and further stresses on how important it is to study this association.

## Review

Irritable bowel syndrome (IBS) is known to affect 21% to 75% of the world's population. Among these statistics, South-East Asia contributes to the least percentage and IBS density seems to be the most in South America [8]. It has shown to affect the female population approximately twice as much as it affects males. Although females are more affected, on the whole, diarrhea-predominant IBS (D-IBS) is more likely to affect males versus constipation-predominant IBS (C-IBS), with abdominal pain and constipation more likely affects females [9]. The spectrum of IBS is more or less uniform for America, with all variants being equally distributed across the continent. However, in Europe, C-IBS and mixed-irritable bowel syndrome (M-IBS) tend to be more prevalent [10]. This review will go forward to discuss the pathophysiology and relationship of C-IBS with methane and how we can use this to our advantage in developing novel drugs that can help combat this life-changing functional gastrointestinal disorder.

Laying the foundation of C-IBS: a functional disorder with constipation

C-IBS has known to affect a great chunk of patients with a functional gut disorder. It is currently defined as per the standard consensus of Rome IV as abdominal pain with hard or lumpy stools characterizing more than 25% of bowel movements and loose or watery stools constituting less than 25% of bowel movements for an average of three days per month over a span of last three months [11]. Over the years, many underlying factors have been linked to the pathophysiology of C-IBS. Among those, changes in the gut microbiota with underlying anti-inflammatory properties, alterations in the level of short-chain fatty acids (SCFAs), defects in serotonin release, genetic makeup supporting polymorphisms of cholecystokinin-1 (CCK1), visceral hypersensitivity, and dyssynergic defecation are a few to mention. Underlying the brick on which treatment modalities are now being developed is the principle of gut transit time [12-16].

Gut transit time and C-IBS

Gut transit time is the time that it takes for stool to pass through the colon. Although it is widely established that slower transit time will most likely compound to constipation, it is not always true. In patients with C-IBS, transit time is slower than in those with chronic constipation in which the defect lies in the sensation of lower transit and not the actual slowing of gut transit in itself [17-18]. Many studies have been conducted to show the effect of gut transit time and constipation. Out of the many factors causing this problem, methane gas has shown to delay transit time [19-20]. Let's first understand the principles of production of methane gas.

Methane biogenesis and gut microbiome

Methane gas is a constituent of human intestinal gas in 30% to 50% of the population. The composition of intestinal gases shows high inter-individual variability and differs in the different anatomical segments of the gut. Intestinal gases are produced mostly in the large intestine because of the fermentation of undigested polysaccharide fraction of carbohydrates. In small intestinal bacterial overgrowth (SIBO), methane production does occur in the small intestine proximal to the mid-jejunum, and this can be detected on methane breath tests [19,21].

Anaerobic methanogens are the predominant contributors of methane in the gut; the rest are contributed by Clostridium and Bacteroides spp. When carbon dioxide and hydrogen gas are released as a result of fermentation and endogenously from cellular oxidation, these anaerobes cause hydrogen to act as an electron donor to reduce carbon dioxide and result in the formation of methane gas with the liberation of -130 kJ/mol of energy, which acts as the sole energy source for methanogens [19]. Among the various phylotypes, there is one particular methanogen that contributes to more than 90% of methane production. Methanobrevibacter smithii, the most dominant methanogen in the gut, is not only fastidious but also difficult to grow in bacterial cultures. Such methanogens contribute to 10% of the gut microbiome and, recently, they have been found in the duodenum, making up about 20% of the total density of duodenal microbiome. Certain individuals produce more methane gas than usual such that, in methane producers, which will be discussed shortly, methanogens can range from 10^7^ to 10^10^ per gram dry weight of feces. The diversity of such flora, in turn, can be studied by a 16S rRNA analysis [21-23]. Results from 16S rRNA-based microbiota profiling approaches demonstrate both quantitative and qualitative changes of mucosal and fecal gut microbiota, particularly in IBS [24].

Role of methane in C-IBS: inert or a lot more - working as a gastrotransmitter?

When fermentation in the gut was previously studied, methane was labeled as an innocent gas with little to do in the gut other than cause flatus and bloating when in excess. Studies were then conducted, which reflected that it is, indeed, much more than just a gas. In 2005, the study of Pimental et al. first showed the influence on methane on small bowel transit, demonstrating that it caused the augmentation of contractile activity of the gut and slowed the peristalsis down. Together with it, also documented was the fact that in patients with IBS, small intestinal contractile activity is increased, making methane the culprit behind the pathogenesis of constipation in IBS. Even though it was clear that methane caused constipation in C-IBS, the proper mechanism was still largely unknown. Given the need for a new treatment modality, in 2017, research was done, which depicted the actions of methane on the intestine being largely influenced by the cholinergic pathway of the enteric nervous system. This was supported mainly by the fact that the methane-induced increase in amplitude was mainly inhibited when one to two hertz of electrical field stimulation (EFS) was applied following atropine infusion. Methane, therefore, significantly increased calcium fluorescence while this increase was attenuated following atropine infusion. EFS and the fluorescence, however, remain unchanged with calcium infusions, tetrodotoxin (TTX), and guanethidine [6,20,25].

Methane production and utility of breath tests: distinguishing methane producers from non-producers

Breath tests have been utilized in C-IBS and SIBO to detect hydrogen and methane levels for many years. Breath test measurements of methane are traditionally obtained with hydrogen via a test called 10-sample lactulose breath test (LBT). In this test, 10 breath samples are obtained every 20 minutes for 180 minutes after feeding 10 g of lactulose substrate. A positive test shows the phenotype of 'methane producers' with the production of 10 parts per million (ppm) of methane in the sample versus below 10 ppm for 'methane nonproducers.' There is a 24-hour preparation period before taking the test; the first 12 hours require a specific diet and the last 12 hours require complete fasting. Other substrates like sorbitol and glucose have also been used [19,26].

Recently, as methane's role in C-IBS is underway, a new test has been specified for measuring methane concentration as a spot-methane breath test. After the consensus of the studies referenced, 5 ppm of methane detected in the spot test is taken as positive for 'methane producers.' Such a test can be obtained with overnight fasting for 12 hours as compared to the 24-hour preparation for LBT. This test has been supported by large national data. Even though this study was formulated especially for SIBO, a growing interest in microbiome manipulation for the treatment of C-IBS will render this test very helpful in carefully selecting patients for specific treatments, which will be directed specifically to methanogenesis [26-27].

Degree of breath methane with different bowel parameters

Recent work has demonstrated a universal association of methane with C-IBS. Methane is not only the culprit for constipation or functional problems of the gut, but it also has shown to quantitatively influence the degree of constipation. IBS patients with detectable methane showed higher constipation severity scores and the quantity of methane seen on the breath test was directly proportional to the degree of constipation reported. This was demonstrated in a detailed nested study, which contrasted constipation severity with methane production status and correlated it with low Bristol stool scores and low bowel movements [28]. One study also linked its detection with the degree of bloating and flatulence, which apparently makes sense as more gas will cause more distension naturally [19,29].

Methane as a potential biomarker of C-IBS

A prospective, double-blind head-to-head comparison of methane positivity on LBT and the Rome I IBS classification showed that methane positivity had a sensitivity of 91.7% and specificity of 81.3% to assigned subjects of C-IBS [30]. Other than this study, a meta-analysis of nine other studies studying 1,277 patients also showed a positive association between C-IBS and methane. Thus, these studies point toward the potential role of methane as a biomarker of C-IBS although it should not be forgotten that it is not the only moiety that is associated with C-IBS [19,31].

Therapeutic approach to methanogenesis: laxatives and antibiotics?

For C-IBS, we have a multitude of medications, ranging from a myriad of laxatives, probiotics, and dietary fibers to antibiotics and centrally acting antidepressants. In order to keep this discussion concise and relevant, we will only probe those drugs that affect methanogenesis directly or indirectly. Methanogens are known to grow well in conditions of slow transit and thus using laxatives will alter the favorable growth environment, leading to relief in C-IBS [32-34].

Antibiotic treatment has gained importance lately. The usage of non-absorbable antibiotics like neomycin and rifaximin has shown proven benefits that were documented in previous studies, including a double-blind placebo controlled-trial study [35-37].

In one study involving neomycin, the elimination of methane on the breath test was designed to study the relief in symptoms. In methane producers, as defined above, improvement in constipation was significantly more as compared to those C-IBS patients who were non-producers [38]. Other than that, neomycin also led to normalization of LBT [35]. A similar double-blind study was also done to investigate rifaximin and showed improved colon transit time and low methane on spot-test in patients who were treated with it [39].

In one study, a combination of neomycin and rifaximin for four weeks proved to be superior to the usage of neomycin alone in alleviating the symptoms of bloating and straining in C-IBS [40].

Targeting HMG-CoA for terminating methanogenesis in C-IBS: new advent in medicine

Currently under research is a prodrug lactone form of lovastatin, which targets methanogenesis directly without increasing antibiotic resistance, as in the case of neomycin. It has also shown to not significantly alter the gut genome. Unlike, the hydroxy acid version of statins that inhibit HMG-CoA for cholesterol synthesis, lactone versions have specific docking capabilities where they are able to inhibit archaeal methanogenesis. Lovastatin is converted to hydroxy acid in the stomach, and absorbed, unlike nonabsorbable antibiotics. To avoid this, trials have worked to innovate galenic form of modified release lovastatin lactone (SYN-010), which has enteric polymers that allow delivery to sites of methanogenesis, i.e. the duodenum and ileocecal region, to transit into the colon [7,41]. This pH-dependent dual-pulse regimen is under an interventional quadruple clinical trial whose results will be published by 2020 with hopes to reduce methanogenesis sustainably and decrease the associated bloating and pain. The advent of statins that will cover up the domain of IBS will increase the quality of life of those affected with C-IBS, with a minimal side-effect profile and better, long-lasting relief.

## Conclusions

Functional gut disorders, particularly constipation-predominant irritable bowel syndrome (C-IBS), have long been known to cause a decrease in the quality of life of patients in all circles. From environmental to organic causes, C-IBS has a lot of factors to tackle. Methane is one such factor, as it decreases the ileal and colon transit time and raises the amplitude of contraction, slowing peristalsis, and resulting in constipation. As described, studies have shown that targeting methanogenesis directly causes relief in the symptoms of C-IBS. Thus diagnostic testing involving the spot-methane test should be made available to classify the methane producer group of patients and to provide a specialized treatment regimen. This will not only be resolute but will also protect the gut flora from developing any sort of dysbiosis and antibiotic resistance. Furthermore, more studies should be conducted to elucidate the role of methane further than what we already know. This new era of microbiome manipulation in neurogastroenterology will provide physicians and research scientists with new ideas and treatment plans to work on.
